# Anterior Nasal Schwannoma: A Rare Sinonasal Neoplasm

**DOI:** 10.7759/cureus.41300

**Published:** 2023-07-03

**Authors:** Eric Freeman, Lauren Hecht, Joel Crum, Matthew Lutz

**Affiliations:** 1 Otolaryngology - Head and Neck Surgery, Western Reserve Hospital, Cuyahoga Falls, USA; 2 Otolaryngology - Head and Neck Surgery, Heritage College of Osteopathic Medicine, Athens, USA; 3 Otolaryngology - Head and Neck Surgery, Lincoln Memorial University-DeBusk College of Osteopathic Medicine, Harrogate, USA

**Keywords:** sinonasal mass, verocay bodies, anterior nasal septum, septal tumor, sinonasal schwannoma

## Abstract

Schwannomas are the most common type of benign peripheral nerve tumor in adults. Schwann cells assist in the conduction of nerve impulses and wrap around peripheral nerves to provide protection and support. Schwannomas typically arise from a single fascicle within the main nerve. Although they can occur anywhere in the body, nasal schwannomas are exceptionally rare.

This case study presents a 65-year-old Caucasian female who had been experiencing obstructive nasal symptoms for three months. The in-office physical examination revealed a soft tissue expansile mass involving the submucosal tissues of the bilateral anterior nasal cavity, located just posterior to the columella. The mass was surgically excised in the operating room, and the diagnosis was confirmed through histopathology.

With only 32 reported cases, nasal septal schwannomas are exceedingly rare. Diagnosis relies on histopathology for confirmation. However, their clinical presentation can mimic other sinonasal pathologies. A septal schwannoma should be considered as a differential diagnosis for a unilateral sinonasal mass. Complete excision is the definitive treatment and is associated with a low recurrence rate. The patient had no signs of reoccurrence on nasal endoscopy three months postoperatively. Surveillance MRI will be completed at one year.

## Introduction

A schwannoma is a benign nerve sheath tumor that can arise from any myelinated nerve fiber. It affects fewer than 200,000 people, and the typical age range of affected individuals is between 50 and 60 years. There is no recognized sex or racial predilection [1]. Head and neck schwannomas account for approximately 25%-40% of all cases. The most common variant in the head and neck region is the vestibular schwannoma, which is associated with cranial nerve eight (CN 8), also known as the vestibulocochlear nerve [2]. The vestibular portion of CN 8 is responsible for carrying information related to balance and spatial orientation from the vestibular apparatus in the inner ear. The cochlear function transmits auditory information from the inner ear to the brain. Only 4% of all schwannomas are associated with the sinonasal tract. Their occurrence is most common in the nasoethmoidial region, followed by the maxillary sinus, frontal sinus, and sphenoid sinus [3,4]. Even more rare are schwannomas of the nasal septum. There are only 32 reported cases of nasal septal schwannomas [5].

## Case presentation

A 65-year-old female was referred to the Ear, Nose, and Throat clinic by her primary care provider for evaluation of a sinonasal mass involving the anterior nasal cavity. The mass was first noticed four months prior and had been progressively enlarging, causing nasal obstruction. She denied any significant pain, rhinorrhea, numbness, epistaxis, anosmia, history of squamous or basal cell carcinoma, or any history of trauma to the area. Her past medical, family, and surgical histories were noncontributory.

On examination, she was noted to have a soft expansile mass involving the submucosal tissues of the bilateral anterior nasal cavity, just posterior to the columella. Initially, the mass appeared to be a cystic lesion. The nasal septum was otherwise midline, with no appreciable mucosal edema or discharge observed during the examination.

The mass moderately obstructed the nasal aperture bilaterally and was increasing in size. Fine-needle aspiration was performed, which yielded no significant fluid or blood. The specimen was sent for pathological cytological interpretation. The cytology report demonstrated rare, atypical cells. Based on the growth velocity observed over a four-month period and the pathological findings, a recommendation was made for intraoperative excision.

**Figure 1:**
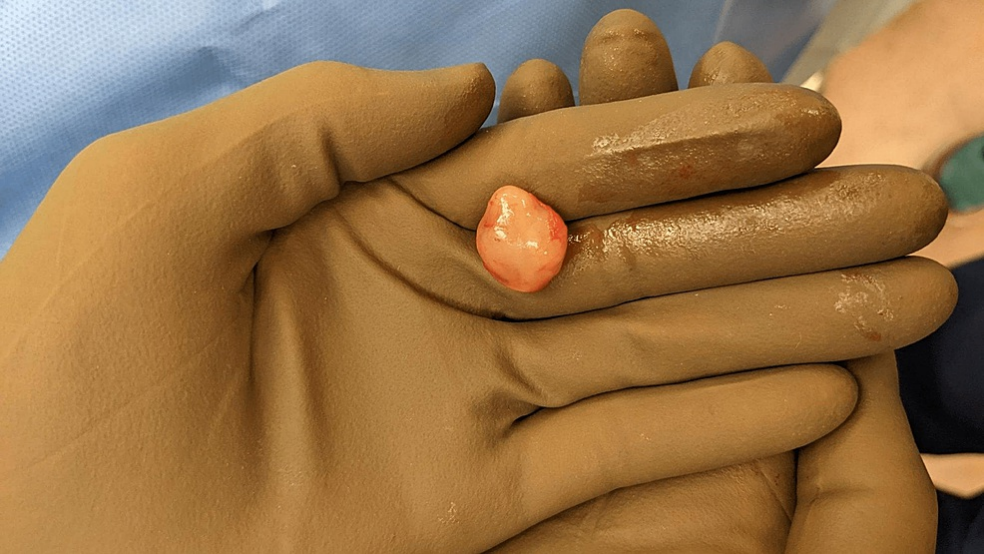
Postoperative nasal schwannoma specimen.

**Figure 2:**
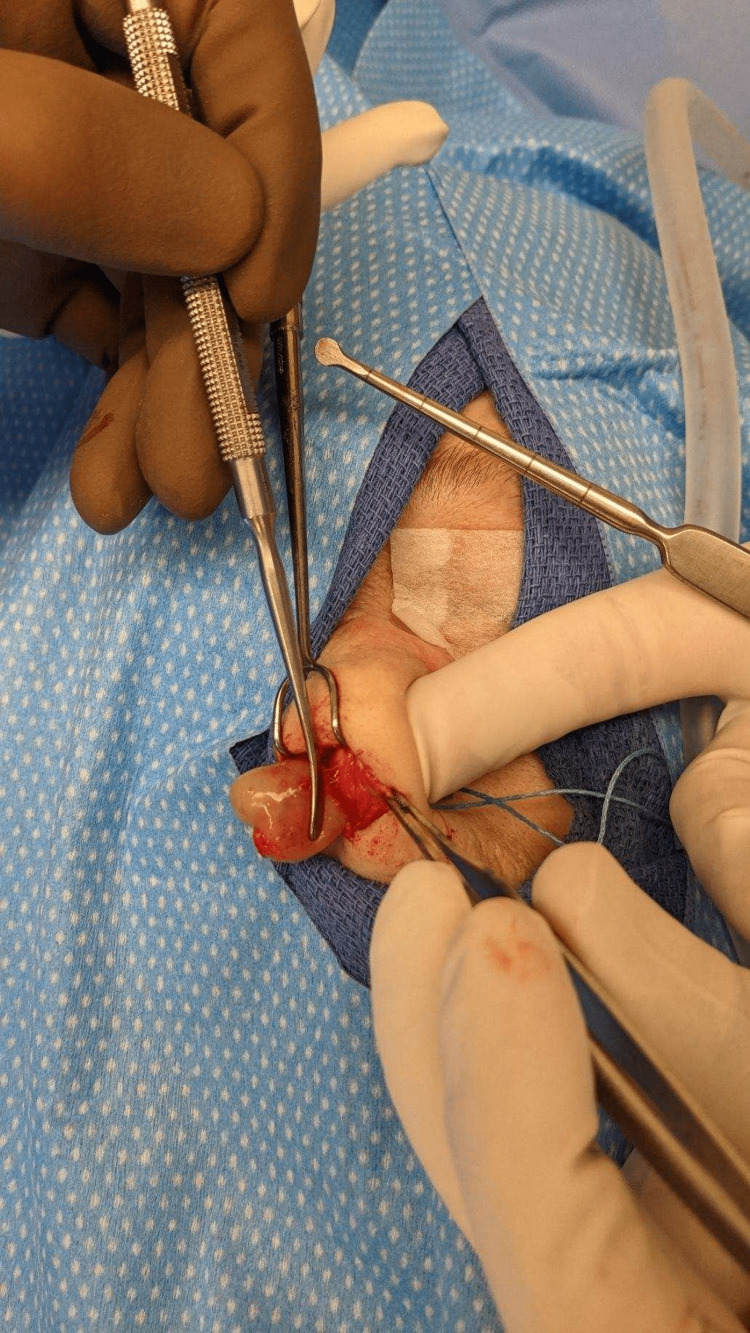
Intraoperative anterior nasal septum schwannoma specimen.

The patient was taken to the operating room for excision of the anterior nasal cavity mass. There was prominent bulging soft tissue involving the anterior nasal cavity bilaterally, just posterior to the columella. The expansile lesion was moderately obstructing the nasal aperture. A pale-colored lobulated soft tissue mass, measuring approximately 2.5 × 1.5 cm in its greatest dimensions (Figure 1), was excised from within the submucoperichondrial plane, primarily involving the left side of the anterior nasal septum (Figure 2). The excised specimen was sent for permanent pathological analysis.

A gross examination of the anterior submucosal mass revealed a 2.1 × 1.7 × 1.1 cm brown-tan, rubbery, polypoid segment of soft tissue. Histological analysis was positive for S100, supporting the diagnosis of a schwannoma.

One month postoperatively, the widening of the anterior septal mucosa had resolved after excision. The right-sided incision had healed, and there was no edema. The patient reported improved nasal obstruction and no cosmetic deformities. There was no evidence of recurrence on nasal endoscopy at her three-month follow-up.

## Discussion

Schwannomas are the most common type of benign peripheral nerve tumors in adults. While 25%-40% of all schwannoma variants occur in the head and neck region, anterior nasal septal schwannomas are exceedingly rare, with only 32 reported cases [5].

Clinically diagnosing a sinonasal schwannoma is difficult due to its vague presenting symptoms and broad differential diagnoses. The most common symptoms of nasal schwannomas include discharge, nasal obstruction, epistaxis, and anosmia [6]. Given the rarity of the tumor and its varied clinical presentation, the differential diagnosis of schwannoma in the nasal and paranasal sinuses includes, but is not limited to, mucocele, inflammatory polyps, angiofibroma, glioma, papilloma, esthesioneuroblastoma, meningioma, sarcoma, squamous cell carcinoma, adenocarcinoma, and lymphomas [7,8].

Due to the wide variety of potential pathologies causing unilateral nasal obstruction, it is challenging to make a diagnosis based solely on imaging. However, MRI imaging sequences are used to aid in the diagnosis of schwannomas. On MRI, schwannomas typically appear as well-circumscribed masses that displace adjacent structures without direct invasion [9]. Common MRI characteristics of schwannomas include enhancement with gadolinium on T1-weighted images and heterogeneously hyperintense areas on T2-weighted images, resulting from the contrast between Antoni Type A and Antoni Type B bodies [10]. Other MRI signs that can assist in the diagnosis include the split fat sign, target sign, and fascicular sign. The split fat sign is represented by a thin peripheral rim of fat, which is best visualized on non-fat-suppressed sequences along the long axis of the lesion. The target sign is characterized by a peripheral high T2 signal and a centrally low signal. The fascicular sign is appreciated when multiple small ring-like structures are observed [11].

The diagnosis of schwannomas can only be confirmed through histopathology [11]. Macroscopically, schwannomas are solid, well-demarcated tumors with an oval, round, or fusiform shape. They have a grayish-to-yellowish color and appear fleshy and shiny on the cut surface [12]. Microscopically, schwannomas are encapsulated tumors with two distinct histological regions. Antoni A tissue exhibits hypercellular spindle cells, sometimes forming palisades around eosinophilic areas known as Verocay bodies. Antoni B tissue demonstrates a hypocellular myxomatous pattern with loose connective tissue. Immunostaining for S100 protein is positive, which further supports the diagnosis [1,8]. Additionally, calretinin and glial fibrillary acidic protein are immunohistochemical stains that can aid in differentiating schwannomas from neurofibromas [13].

As in this case, the treatment of choice for schwannomas is complete surgical excision. Depending on the tumor location, surgical excision can be achieved through endoscopic endonasal surgery or lateral rhinotomy. The endoscopic approach is considered the standard surgical approach due to its ability to visualize the mass and avoid external incisions. The need for intraoperative imaging depends on the location of the schwannoma [14].

Recurrence of schwannomas is rare, but it is important to monitor patients in the short and long term. In this case, the patient did not experience recurrence at the three-month follow-up. An MRI is scheduled for one-year post-operation to evaluate for any signs of recurrence [15].

## Conclusions

Nasal schwannomas are very rare. Their presenting symptoms are common sinonasal complaints, making the differential diagnosis extensive. The office workup typically includes nasal endoscopy and/or fine-needle aspiration, depending on the location of the mass. A preoperative MRI can confirm whether the mass is of neurologic origin. Diagnosis requires surgical excision and histological evaluation. Endoscopic endonasal surgery is the standard treatment for nasal schwannomas. These tumors are benign masses with low recurrence rates following excision, and resection is considered curative. Surveillance with MRI at one, five, and ten years postoperatively is recommended for monitoring.
